# The genome sequence of the Marbled Minor moth, *Oligia strigilis *(Linnaeus, 1758)

**DOI:** 10.12688/wellcomeopenres.21679.1

**Published:** 2024-05-21

**Authors:** Gavin R. Broad, Natural History Museum Genome Acquisition Lab, Darwin Tree of Life Barcoding collective, Wellcome Sanger Institute Tree of Life Management, Samples and Laboratory team, Wellcome Sanger Institute Scientific Operations: Sequencing Operations, Wellcome Sanger Institute Tree of Life Core Informatics team, Tree of Life Core Informatics collective, Darwin Tree of Life Consortium

**Affiliations:** 1 Natural History Museum, London, England, UK

**Keywords:** Oligia strigilis, Marbled Minor moth, genome sequence, chromosomal, Lepidoptera

## Abstract

We present a genome assembly from an individual male *Oligia strigilis* (Marbled Minor; Arthropoda; Insecta; Lepidoptera; Noctuidae). The genome sequence is 626.1 megabases in span. Most of the assembly is scaffolded into 31 chromosomal pseudomolecules, including the Z sex chromosome. The mitochondrial genome has also been assembled and is 15.35 kilobases in length.

## Species taxonomy

Eukaryota; Opisthokonta; Metazoa; Eumetazoa; Bilateria; Protostomia; Ecdysozoa; Panarthropoda; Arthropoda; Mandibulata; Pancrustacea; Hexapoda; Insecta; Dicondylia; Pterygota; Neoptera; Endopterygota; Amphiesmenoptera; Lepidoptera; Glossata; Neolepidoptera; Heteroneura; Ditrysia; Obtectomera; Noctuoidea; Noctuidae; Noctuinae; Apameini; *Oligia*; *Oligis strigilis* (Linnaeus, 1758) (NCBI:txid988009).

## Background

*Oligis strigilis*, Marbled Minor, is one of a trio of very similar *Oligia* species in the UK. To safely identify *O. strigilis, O. latruncula* or *O. versicolor*, genitalia should be examined. Marbled Minor tends, as its name suggests, to be particularly contrastingly marked with white and brown/black, but there is much overlap in appearance with other species, and melanic forms are frequent in all three. Male genitalia are distinctive, with a long, thin harpe (or ‘clasper’) in *O. strigilis*, while females are identified by the sharply narrowed junction of the ductus bursae and antrum (see Townsend *et al.*, 2010). The genome assembly is from a male, with the identification confirmed by genitalia examination as well as by DNA barcoding. South ( South, 1907), in his very influential volumes on British moths (which GRB used extensively when beginning light trapping, although he is not quite that old), treated the three as one species, Marbled Minor, and they are frequently lumped together now for recording, as a species aggregate.

Found over much of Britain, but scarce in Ireland ( Bond, 2005), *Oligia strigilis* feeds as a larva in grass stems, moving between stems ( Waring *et al.*, 2017). It is found throughout Europe, as far east as Central Asia, and has been accidentally introduced to North America where it has spread rapidly in the North-east ( Handfield, 1999; Lafontaine & Schmidt, 2010). As with various other species of grass-feeding moths, the abundance of *O. strigilis* has decreased markedly in Britain, by 76% since the 1970s, although its distribution has potentially increased ( Randle *et al.*, 2019). In contrast, in Germany and Romania *O. strigilis* has been reported to periodically become a pest of Cock’s-foot Grass ( *Dactylis glomerata*), which is an important forage species ( Mateias & Dӑnulescu, 1990; Stechow, 1926).

As with various other moth species, most famously Peppered Moth ( *Biston betularia*), the distribution and frequency of melanism in *O. strigilis* has been of great interest. Mikkola (1980) suggested that, at least in Finland, patterns of melanism in *O. strigilis* were rather different to patterns in *O. latruncula*, with melanism apparently having evolved independently in different locations in *O. strigilis*. Genomes of closely related species, such as *Oligia* species, and more distantly related Lepidoptera, will enable new insights into the evolution of melanism and other important topics.

## Genome sequence report

**Figure 1.**
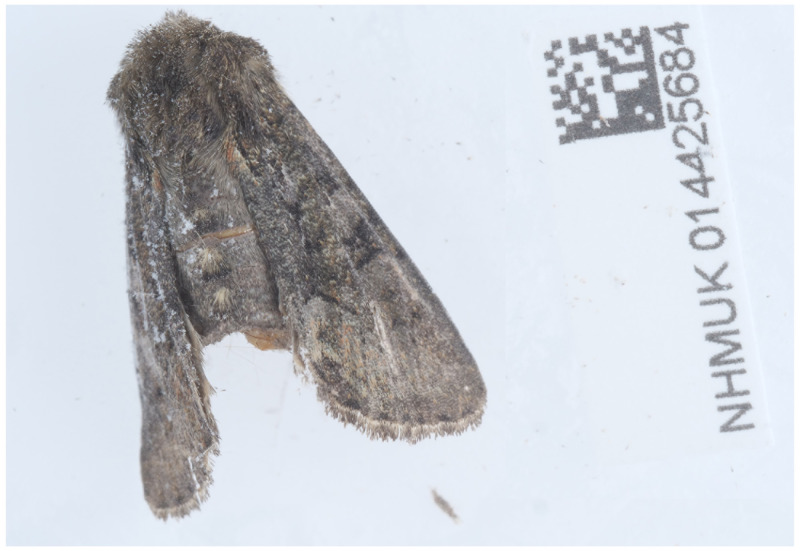
Photograph of the *Oligia strigilis* (ilOliStri1) specimen used for genome sequencing.

The genome was sequenced from a male *Oligia strigilis* ( Figure 1) collected from Tonbridge, Kent, UK (51.19, 0.29). A total of 26-fold coverage in Pacific Biosciences single-molecule HiFi long reads was generated. Primary assembly contigs were scaffolded with chromosome conformation Hi-C data. Manual assembly curation corrected 308 missing joins or mis-joins and removed 15 haplotypic duplications, reducing the assembly length by 0.43% and the scaffold number by 8.02%, and increasing the scaffold N50 by 3.60%.

**Figure 2.**
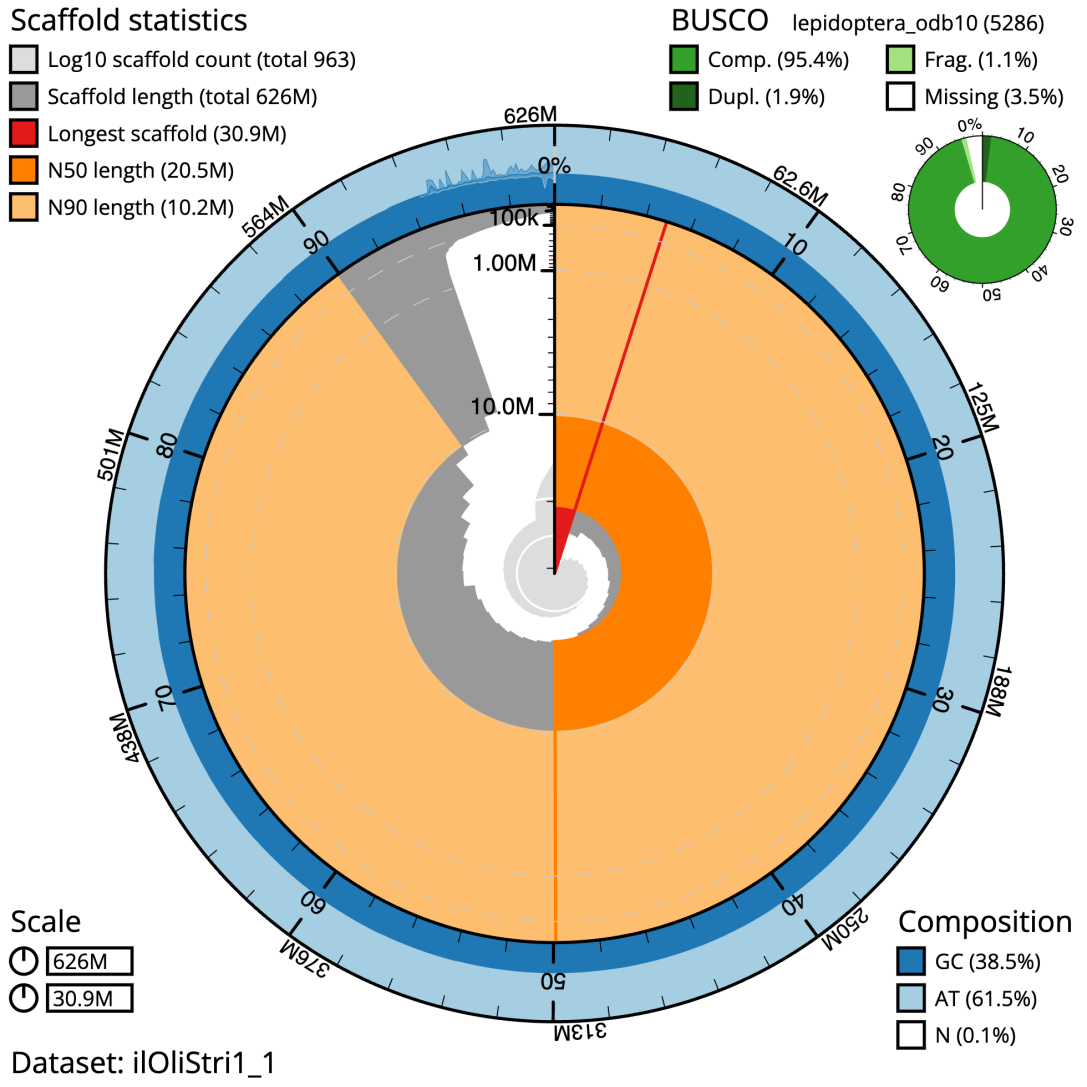
Genome assembly of *Oligia strigilis*, ilOliStri1.1: metrics. The BlobToolKit snail plot shows N50 metrics and BUSCO gene completeness. The main plot is divided into 1,000 size-ordered bins around the circumference with each bin representing 0.1% of the 626,116,773 bp assembly. The distribution of scaffold lengths is shown in dark grey with the plot radius scaled to the longest scaffold present in the assembly (30,867,984 bp, shown in red). Orange and pale-orange arcs show the N50 and N90 scaffold lengths (20,522,485 and 10,164,546 bp), respectively. The pale grey spiral shows the cumulative scaffold count on a log scale with white scale lines showing successive orders of magnitude. The blue and pale-blue area around the outside of the plot shows the distribution of GC, AT and N percentages in the same bins as the inner plot. A summary of complete, fragmented, duplicated and missing BUSCO genes in the lepidoptera_odb10 set is shown in the top right. An interactive version of this figure is available at https://blobtoolkit.genomehubs.org/view/ilOliStri1_1/dataset/ilOliStri1_1/snail.

**Figure 3.**
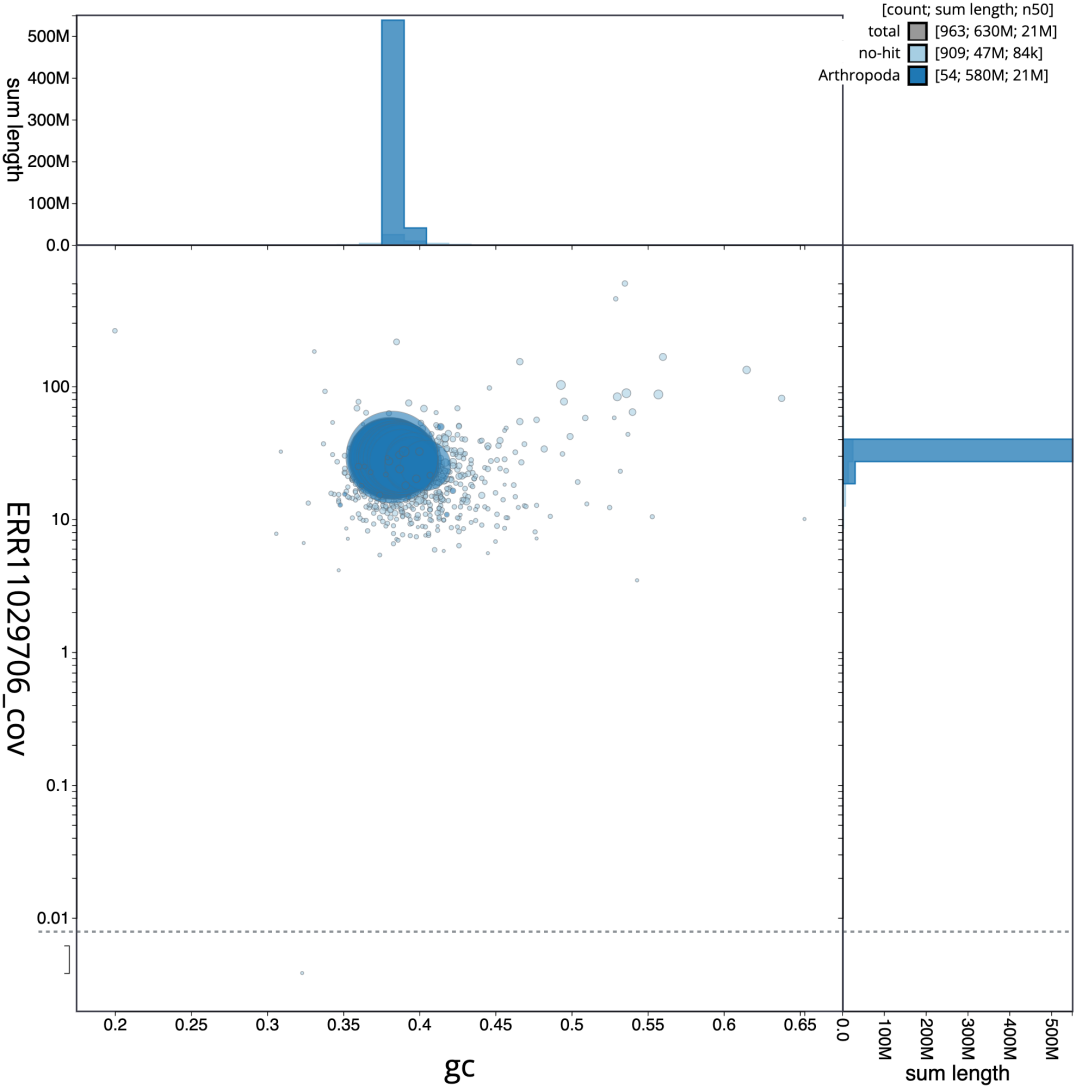
Genome assembly of *Oligia strigilis*, ilOliStri1.1: BlobToolKit GC-coverage plot. Sequences are coloured by phylum. Circles are sized in proportion to sequence length. Histograms show the distribution of sequence length sum along each axis. An interactive version of this figure is available at https://blobtoolkit.genomehubs.org/view/ilOliStri1_1/dataset/ilOliStri1_1/blob.

**Figure 4.**
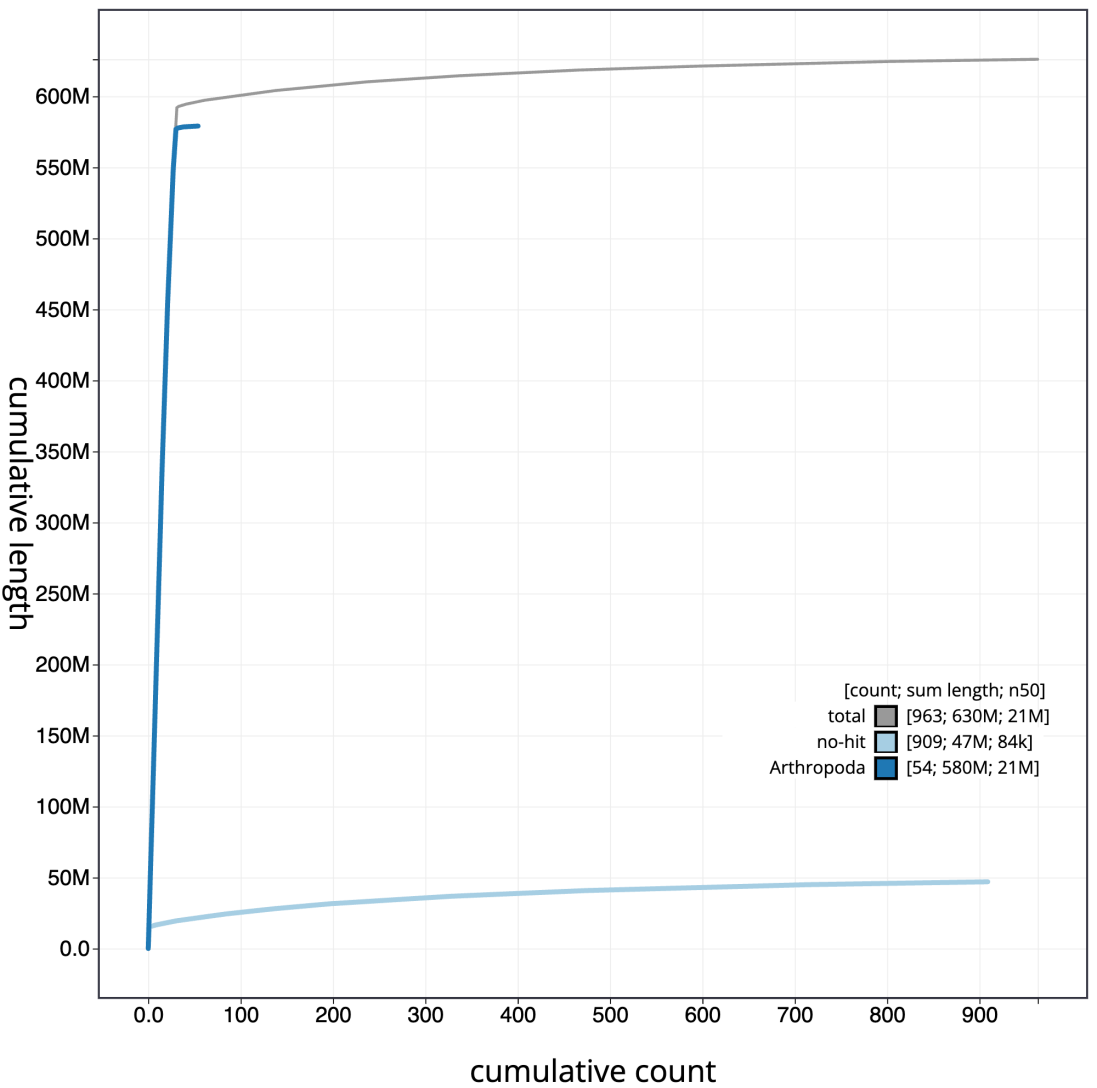
Genome assembly of *Oligia strigilis*, ilOliStri1.1: BlobToolKit cumulative sequence plot. The grey line shows cumulative length for all sequences. Coloured lines show cumulative lengths of sequences assigned to each phylum using the buscogenes taxrule. An interactive version of this figure is available at https://blobtoolkit.genomehubs.org/view/ilOliStri1_1/dataset/ilOliStri1_1/cumulative.

**Figure 5.**
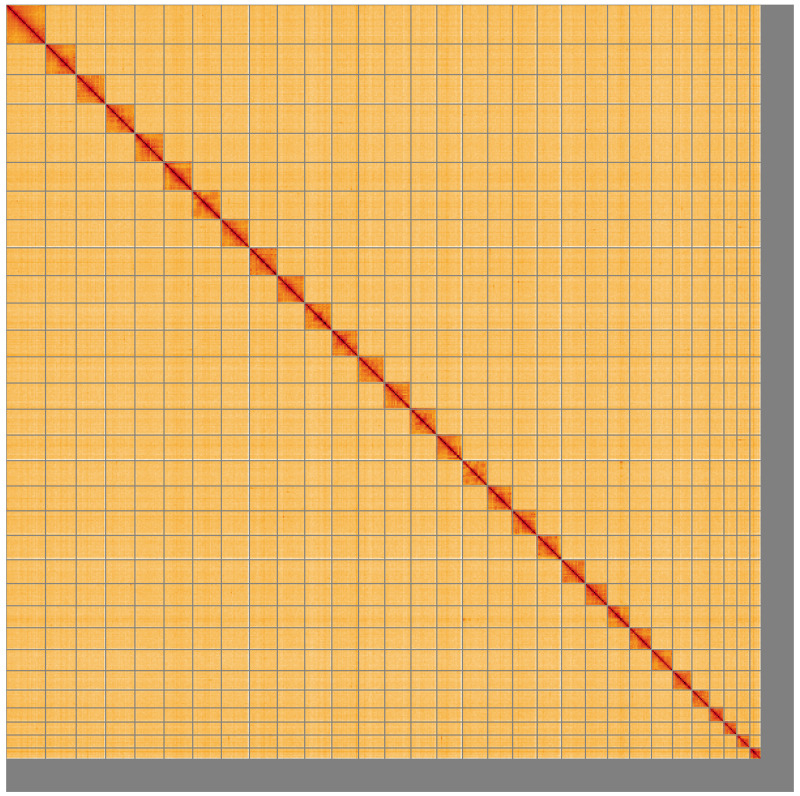
Genome assembly of *Oligia strigilis*, ilOliStri1.1: Hi-C contact map of the ilOliStri1.1 assembly, visualised using HiGlass. Chromosomes are shown in order of size from left to right and top to bottom. An interactive version of this figure may be viewed at https://genome-note-higlass.tol.sanger.ac.uk/l/?d=TIJpB9F5TuGoVC-dH_GAGA.

The final assembly has a total length of 626.1 Mb in 962 sequence scaffolds with a scaffold N50 of 20.5 Mb ( Table 1). The snail plot in Figure 2 provides a summary of the assembly statistics, while the distribution of assembly scaffolds on GC proportion and coverage is shown in Figure 3. The cumulative assembly plot in Figure 4 shows curves for subsets of scaffolds assigned to different phyla. Most (95%) of the assembly sequence was assigned to 31 chromosomal-level scaffolds, representing 30 autosomes and the Z sex chromosome. Chromosome-scale scaffolds confirmed by the Hi-C data are named in order of size ( Figure 5; Table 2). The Z chromosome was assigned based on synteny to *Oligia latruncula* (GCA_948474745.1) ( Broad *et al.*, 2023). While not fully phased, the assembly deposited is of one haplotype. Contigs corresponding to the second haplotype have also been deposited. The mitochondrial genome was also assembled and can be found as a contig within the multifasta file of the genome submission.

**Table 1.** Genome data for *Oligia strigilis*, ilOliStri1.1.

Project accession data		
Assembly identifier	ilOliStri1.1	
Species	*Oligia strigilis*	
Specimen	ilOliStri1	
NCBI taxonomy ID	988009	
BioProject	PRJEB60724	
BioSample ID	SAMEA11025011	
Isolate information	ilOliStri1, male: head and thorax (DNA sequencing) ilOliStri2, male: head and thorax (Hi-C sequencing), abdomen (RNA)	
Assembly metrics *		*Benchmark*
Consensus quality (QV)	57.0	*≥ 50*
*k*-mer completeness	99.99%	*≥ 95%*
BUSCO **	C:95.4%[S:93.5%,D:1.9%], F:1.1%,M:3.5%,n:5,286	*C ≥ 95%*
Percentage of assembly mapped to chromosomes	95%	*≥ 95%*
Sex chromosomes	Z	*localised homologous pairs*
Organelles	Mitochondrial genome: 15.35 kb	*complete single alleles*
Raw data accessions		
PacificBiosciences SEQUEL II	ERR11029706	
Hi-C Illumina	ERR11042968	
PolyA RNA-Seq Illumina	ERR12708745	
Genome assembly		
Assembly accession	GCA_951800025.1	
*Accession of alternate haplotype*	GCA_951800045.1	
Span (Mb)	626.1	
Number of contigs	3345	
Contig N50 length (Mb)	0.4	
Number of scaffolds	962	
Scaffold N50 length (Mb)	20.5	
Longest scaffold (Mb)	30.87	

* Assembly metric benchmarks are adapted from column VGP-2020 of “Table 1: Proposed standards and metrics for defining genome assembly quality” from Rhie *et al.* (2021). ** BUSCO scores based on the lepidoptera_odb10 BUSCO set using version 5.3.2. C = complete [S = single copy, D = duplicated], F = fragmented, M = missing, n = number of orthologues in comparison. A full set of BUSCO scores is available at https://blobtoolkit.genomehubs.org/view/ilOliStri1_1/dataset/ilOliStri1_1/busco.

**Table 2.** Chromosomal pseudomolecules in the genome assembly of *Oligia strigilis*, ilOliStri1.

INSDC accession	Chromosome	Length (Mb)	GC%
OX637541.1	1	24.02	38.0
OX637542.1	2	22.43	38.5
OX637543.1	3	23.11	38.0
OX637544.1	4	22.95	38.0
OX637545.1	5	22.83	38.0
OX637546.1	6	22.52	38.5
OX637547.1	7	22.07	38.0
OX637548.1	8	21.82	38.0
OX637549.1	9	21.52	38.0
OX637550.1	10	21.25	38.0
OX637551.1	11	20.95	38.5
OX637552.1	12	20.52	38.0
OX637553.1	13	20.57	38.0
OX637554.1	14	20.33	38.0
OX637555.1	15	20.08	38.5
OX637556.1	16	19.85	38.5
OX637557.1	17	19.5	38.5
OX637558.1	18	19.11	38.5
OX637559.1	19	19.28	38.5
OX637560.1	20	18.71	39.0
OX637561.1	21	17.38	38.5
OX637562.1	22	17.26	39.0
OX637563.1	23	17.14	38.5
OX637564.1	24	16.54	39.0
OX637565.1	25	15.18	38.5
OX637566.1	26	14.01	39.0
OX637567.1	27	11.13	39.5
OX637568.1	28	10.16	39.5
OX637569.1	29	10.16	39.5
OX637570.1	30	8.79	40.5
OX637540.1	Z	30.87	38.0
OX637571.1	MT	0.02	20.0

The estimated Quality Value (QV) of the final assembly is 57.0 with *k*-mer completeness of 99.99%, and the assembly has a BUSCO v5.3.2 completeness of 95.4% (single = 93.5%, duplicated = 1.9%), using the lepidoptera_odb10 reference set ( *n* = 5,286).

Metadata for specimens, barcode results, spectra estimates, sequencing runs, contaminants and pre-curation assembly statistics are given at https://links.tol.sanger.ac.uk/species/988009.

## Methods

### Sample acquisition and nucleic acid extraction

A male *Oligia strigilis* (specimen ID NHMUK014425684, ToLID ilOliStri1) was collected from Tonbridge, Kent, UK (latitude 51.19, longitude 0.29) on 2021-07-02 in a light trap. The specimen used for Hi-C and RNA sequencing (specimen ID NHMUK014425677, ToLID ilOliStri2), also a male, was collected from the same location on 2021-06-25 in a light trap. The specimens were collected and identified by Gavin Broad (Natural History Museum) and preserved by dry freezing at –80°C.

The workflow for high molecular weight (HMW) DNA extraction at the Wellcome Sanger Institute (WSI) includes a sequence of core procedures: sample preparation; sample homogenisation, DNA extraction, fragmentation, and clean-up. The sample was prepared for extraction at the WSI Tree of Life Core Laboratory: the ilOliStri1 sample was weighed and dissected on dry ice ( Jay *et al.*, 2023). Tissue from the head and thorax was homogenised using a PowerMasher II tissue disruptor ( Denton *et al.*, 2023a). HMW DNA was extracted in the WSI Scientific Operations core using the Automated MagAttract v2 protocol ( Oatley *et al.*, 2023). The DNA was sheared into an average fragment size of 12–20 kb in a Megaruptor 3 system with speed setting 31 ( Bates *et al.*, 2023). Sheared DNA was purified by solid-phase reversible immobilisation ( Strickland *et al.*, 2023): in brief, the method employs a 1.8X ratio of AMPure PB beads to sample to eliminate shorter fragments and concentrate the DNA. The concentration of the sheared and purified DNA was assessed using a Nanodrop spectrophotometer and Qubit Fluorometer and Qubit dsDNA High Sensitivity Assay kit. Fragment size distribution was evaluated by running the sample on the FemtoPulse system.

RNA was extracted from abdomen tissue of ilOliStr2 in the Tree of Life Laboratory at the WSI using the RNA Extraction: Automated MagMax™ *mir*Vana protocol ( do Amaral *et al.*, 2023). The RNA concentration was assessed using a Nanodrop spectrophotometer and a Qubit Fluorometer using the Qubit RNA Broad-Range Assay kit. Analysis of the integrity of the RNA was done using the Agilent RNA 6000 Pico Kit and Eukaryotic Total RNA assay.

Protocols developed by the WSI Tree of Life laboratory are publicly available on protocols.io ( Denton *et al.*, 2023b).

### Sequencing

Pacific Biosciences HiFi circular consensus DNA sequencing libraries were constructed according to the manufacturers’ instructions. Poly(A) RNA-Seq libraries were constructed using the NEB Ultra II RNA Library Prep kit. DNA and RNA sequencing was performed by the Scientific Operations core at the WSI on Pacific Biosciences SEQUEL II (HiFi) and Illumina NovaSeq 6000 (RNA-Seq) instruments. Hi-C data were also generated from head and thorax tissue of ilOliStri2 using the Arima2 kit and sequenced on the Illumina NovaSeq 6000 instrument.

### Genome assembly and curation

Assembly was carried out with Hifiasm ( Cheng *et al.*, 2021) and haplotypic duplication was identified and removed with purge_dups ( Guan *et al.*, 2020). The assembly was then scaffolded with Hi-C data ( Rao *et al.*, 2014) using YaHS ( Zhou *et al.*, 2023). The assembly was checked for contamination and corrected using the full version of the TreeVal pipeline ( Pointon *et al.*, 2023). Manual curation was performed using JBrowse2 ( Diesh *et al.*, 2023), HiGlass ( Kerpedjiev *et al.*, 2018) and PretextView ( Harry, 2022). The mitochondrial genome was assembled using MitoHiFi ( Uliano-Silva *et al.*, 2023), which runs MitoFinder ( Allio *et al.*, 2020) or MITOS ( Bernt *et al.*, 2013) and uses these annotations to select the final mitochondrial contig and to ensure the general quality of the sequence.

### Evaluation of the final assembly

A Hi-C map for the final assembly was produced using bwa-mem2 ( Vasimuddin *et al.*, 2019) in the Cooler file format ( Abdennur & Mirny, 2020). To assess the assembly metrics, the *k*-mer completeness and QV consensus quality values were calculated in Merqury ( Rhie *et al.*, 2020). This work was done using Nextflow ( Di Tommaso *et al.*, 2017) DSL2 pipelines “sanger-tol/readmapping” ( Surana *et al.*, 2023a) and “sanger-tol/genomenote” ( Surana *et al.*, 2023b). The genome was analysed within the BlobToolKit environment ( Challis *et al.*, 2020) and BUSCO scores ( Manni *et al.*, 2021; Simão *et al.*, 2015) were calculated.

Table 3 contains a list of relevant software tool versions and sources.

**Table 3.** Software tools: versions and sources.

Software tool	Version	Source
BlobToolKit	4.2.1	https://github.com/blobtoolkit/blobtoolkit
BUSCO	5.3.2	https://gitlab.com/ezlab/busco
Hifiasm	0.16.1-r375	https://github.com/chhylp123/hifiasm
HiGlass	1.11.6	https://github.com/higlass/higlass
Merqury	MerquryFK	https://github.com/thegenemyers/MERQURY.FK
MitoHiFi	2	https://github.com/marcelauliano/MitoHiFi
PretextView	0.2	https://github.com/wtsi-hpag/PretextView
purge_dups	1.2.3	https://github.com/dfguan/purge_dups
sanger-tol/genomenote	v1.0	https://github.com/sanger-tol/genomenote
sanger-tol/readmapping	1.1.0	https://github.com/sanger-tol/readmapping/tree/1.1.0
TreeVal	1.0.0.	https://github.com/sanger-tol/treeval
YaHS	yahs-1.1.91eebc2	https://github.com/c-zhou/yahs

### Wellcome Sanger Institute – Legal and Governance

The materials that have contributed to this genome note have been supplied by a Darwin Tree of Life Partner. The submission of materials by a Darwin Tree of Life Partner is subject to the **‘Darwin Tree of Life Project Sampling Code of Practice’**, which can be found in full on the Darwin Tree of Life website here. By agreeing with and signing up to the Sampling Code of Practice, the Darwin Tree of Life Partner agrees they will meet the legal and ethical requirements and standards set out within this document in respect of all samples acquired for, and supplied to, the Darwin Tree of Life Project.

Further, the Wellcome Sanger Institute employs a process whereby due diligence is carried out proportionate to the nature of the materials themselves, and the circumstances under which they have been/are to be collected and provided for use. The purpose of this is to address and mitigate any potential legal and/or ethical implications of receipt and use of the materials as part of the research project, and to ensure that in doing so we align with best practice wherever possible. The overarching areas of consideration are:

•     Ethical review of provenance and sourcing of the material

•     Legality of collection, transfer and use (national and international)

Each transfer of samples is further undertaken according to a Research Collaboration Agreement or Material Transfer Agreement entered into by the Darwin Tree of Life Partner, Genome Research Limited (operating as the Wellcome Sanger Institute), and in some circumstances other Darwin Tree of Life collaborators.

## Data Availability

European Nucleotide Archive: *Oligia strigilis*. Accession number PRJEB60724; https://identifiers.org/ena.embl/PRJEB60724 ( Wellcome Sanger Institute, 2023). The genome sequence is released openly for reuse. The *Oligia strigilis* genome sequencing initiative is part of the Darwin Tree of Life (DToL) project. All raw sequence data and the assembly have been deposited in INSDC databases. The genome will be annotated using available RNA-Seq data and presented through the Ensembl pipeline at the European Bioinformatics Institute. Raw data and assembly accession identifiers are reported in Table 1.

## Author information

Members of the Natural History Museum Genome Acquisition Lab are listed here: https://doi.org/10.5281/zenodo.7139035.

Members of the Darwin Tree of Life Barcoding collective are listed here: https://doi.org/10.5281/zenodo.4893703.

Members of the Wellcome Sanger Institute Tree of Life Management, Samples and Laboratory team are listed here: https://doi.org/10.5281/zenodo.10066175.

Members of Wellcome Sanger Institute Scientific Operations: Sequencing Operations are listed here: https://doi.org/10.5281/zenodo.10043364.

Members of the Wellcome Sanger Institute Tree of Life Core Informatics team are listed here: https://doi.org/10.5281/zenodo.10066637.

Members of the Tree of Life Core Informatics collective are listed here: https://doi.org/10.5281/zenodo.5013541.

Members of the Darwin Tree of Life Consortium are listed here: https://doi.org/10.5281/zenodo.4783558.
