# Supplementary material for: The genome sequence of the Marbled Minor moth, Oligia strigilis (Linnaeus, 1758)
Source: Wellcome Open Res. 2024 May 21;9:282. [Version 1] doi: 10.12688/wellcomeopenres.21679.1 (PMC11437290; doi:10.12688/wellcomeopenres.21679.1)
Supplement: Peer review history

# Reviewer report: Reviewer response for version 1

**DOI:** 10.21956/wellcomeopenres.23952.r97617

**Published:** 2024-09-27

**Authors:** Leonardo Barbosa Koerich

**Affiliations:** 1 Federal University of Minas Gerais, State of Minas Gerais, Brazil

The manuscript describes the genome assembly of *Oligia strigilis*, also known as the Marbled Minor, a moth species closely related to *O. latruncula* and *O. versicolor*. These three species are morphologically similar, often necessitating genitalia examination or DNA barcoding for accurate identification. This study sequenced the genome of *O. strigilis* using Pacific Biosciences HiFi long reads, supplemented by Hi-C data for chromosome-level scaffolding. The genome assembly covered 626.1 Mb, with a scaffold N50 of 20.5 Mb, representing 95% of the sequence assigned to 31 chromosomal scaffolds, including the Z sex chromosome. BUSCO completeness reached 95.4% suggesting a high-quality genome assembly. Unfortunately, the authors did not annotate any coding genes and the number of coding sequences in *O. strigilis* remains unknown.

 This genome assembly enables further exploration into the evolution of biological traits in Lepidoptera. It also contributes to understanding the genetics of grass-feeding moth species, which are essential both ecologically and agriculturally.

Are sufficient details of methods and materials provided to allow replication by others?

Yes

Is the rationale for creating the dataset(s) clearly described?

Yes

Are the datasets clearly presented in a useable and accessible format?

Yes

Are the protocols appropriate and is the work technically sound?

Yes

Reviewer Expertise:

Structural Genomics and Evolutive Genomics

I confirm that I have read this submission and believe that I have an appropriate level of expertise to confirm that it is of an acceptable scientific standard.

# Reviewer report: Reviewer response for version 1

**DOI:** 10.21956/wellcomeopenres.23952.r95456

**Published:** 2024-09-02

**Authors:** Joana Isabel Meier

**Affiliations:** 1 Department of Zoology, University of Cambridge, Cambridge, England, UK

This genome note provides information on a newly created chromosome-level genome assembly of *Oligia strigilis* (Marbled Minor). There is an informative introduction showing that the genome is useful to study melanism in this group of multiple closely related moth species.

 The methods are detailed and the figures show clearly that the genome is of excellent quality with no evidence of contamination (Fig. 3), high contiguity (Fig. 4) and no evidence of misassemblies (Fig. 5). The genome contains 31 chromosomes like most Lepidoptera and it is publicly accessible.

Are sufficient details of methods and materials provided to allow replication by others?

Yes

Is the rationale for creating the dataset(s) clearly described?

Yes

Are the datasets clearly presented in a useable and accessible format?

Yes

Are the protocols appropriate and is the work technically sound?

Yes

Reviewer Expertise:

evolutionary genomics, with a focus on Lepidoptera, spiders, cichlid fishes and lizards.

I confirm that I have read this submission and believe that I have an appropriate level of expertise to confirm that it is of an acceptable scientific standard.
